# The learning curve for minimally invasive Achilles repair using the “lumbar puncture needle and oval forceps” technique

**DOI:** 10.1186/s12891-024-07489-9

**Published:** 2024-05-11

**Authors:** Yanrui Zhao, Hanzhou Wang, Binzhi Zhao, Shuo Diao, Yuling Gao, Junlin Zhou, Yang Liu

**Affiliations:** Department of Orthopedic Surgery, Beijing Chaoyang Hospital, Capital Medical University, 8 Gongren Tiyuchang Nanlu, Chaoyang District, 100020 Beijing, P.R. China

**Keywords:** Acute Achilles tendon rupture, Clinical outcomes, Learning curves, Minimally invasive

## Abstract

**Introduction:**

An acute Achilles tendon rupture represents a common tendon injury, and its operative methods have been developed over the years. This study aimed to quantify the learning curve for the minimally invasive acute Achilles tendon rupture repair.

**Methods:**

From May 2020 to June 2022, sixty-seven patient cases who received minimally invasive tendon repair were reviewed. Baseline data and operative details were collected. The cumulative summation (CUSUM) control chart was used for the learning curve analyses. Achilles tendon rupture score (ATRS), American Orthopedic Foot and Ankle Society (AOFAS) ankle/hindfoot score, and visual analog scale (VAS) at 3/6/9/12 months were calculated to assess the clinical outcomes.

**Results:**

Thirty-six cases underwent at least a year of follow up and were enrolled in this study. The gender ratio and average age were 80.5% and 32.5 years. The linear equation fitted well (R^2^ = 0.95), and CUSUM for operative time peaked in the 12th case, which was divided into the learning phase (n = 12) and master phase (n = 24). No significant difference was detected between the two groups in clinical variables, except for the operative time (71.1 ± 13.2 min vs 45.8 ± 7.2 min, p = 0.004). Moreover, we detected one case with a suture reaction and treated it properly.

**Conclusion:**

Minimally invasive Achilles repair provides an opportunity for early rehabilitation. Notably, the learning curve showed that the “lumbar puncture needle and oval forceps” technique was accessible to surgeons.

## Background

The Achilles tendon is the strongest and largest tendon in the human body, and its rupture is a common sports-related injury [1, 2]. The incidence of acute Achilles tendon rupture keeps rising, and it has been reported to reach 18 per 100,000 people per year due to improper strength explosions in the ankle or the weakening of elasticity during frequent and high-intensity physical exercise [3–5]. Most acute Achilles tendon ruptures are observed in middle-aged men, predominantly during sports activities that require abrupt initiation and cessation of movement, including tennis, basketball, soccer, and badminton [6]. Notably, between 81 and 89% of rupture cases are attributed to the physical demands of athletic activities as the primary cause of injury [7–9]. Acute Achilles tendon rupture usually requires operative intervention. Otherwise, passive therapy may cause continuous pain and limited motion of the overall ankle joint and even leave a lifelong disability due to the extremity atrophy of the injured side.

Notably, this operative procedure has varied over the years. As such, the open repair requires a 10—15 cm incision to broadly separate the subcutaneous fascia and tendon sheath to search the broken ends for tendon anastomosis. This method was performed under direct supervision and could effectively protect the sural nerve, which was reported to significantly improve clinical outcomes compared with nonoperative options [10]. In 1977, Prof Ma and Griffith introduced the mini-open technique for acute Achilles tendon ruptures. They reported that this advanced attempt has many advantages, including a rapid recovery, a short hospitalization period, and a cosmetic appearance at the surgical site [11]. Recent literature also reported a series of surgical methods for minimally invasive approaches and described their clinical effects to minimize adverse events [12–14]. An Achilles tendon rupture guideline indicates that acute Achilles tendon rupture is a suitable indication for minimally invasive techniques, with clinical recovery rates of at least 85% [15]. Nevertheless, many institutions lack professional facilities to perform this surgery, and the importation of some equipment is limited for financial and administrative reasons.

In recent years, we have referred to previous reports that used the “lumbar puncture needle and oval forceps” technique to repair the ruptured Achilles tendon [16–18]. Generally, the surgical proficiency of orthopedic surgeons could be assessed using statistical analysis of surgical details, radiographic parameters, and postoperative clinical outcomes. However, this traditional evaluation has shown limited accuracy in demonstrating a surgeon’s ability. As such, the learning curve can be used to measure competency and proficiency in various surgical procedures [19, 20]. Notably, this progressive method has been widely used to estimate foot and ankle surgery [21, 22].

Based on this, our study used the cumulative sum (CUSUM) methodology to present the learning process of this minimally invasive operation in treating acute Achilles tendon rupture.

## Methods

### Study design

With the approval of the institutional review committee, a retrospective study was performed on patients who underwent operative treatments for acute Achilles tendon ruptures in 36 from May 2020 to June 2022.

Inclusion criteria were as follows: (1) patients over 18 years old, (2) within two weeks after injury, (3) the definitive diagnosis of the Achilles tendon rupture confirmed by preoperative ultrasound and MRI, (4) positive results of Thompson test and the palpable gap at the rupture sites of Achilles tendon, (5) the Achilles tendon rupture occurring at 2–8 cm proximal to insertion (non-insertional type), (6) receiving a minimally invasive treatment of “lumbar puncture needle and oval forceps,” (7) follow-up more than twelve months. Exclusion criteria: (1) patients under 18 years old, (2) chronic injury (> 2 weeks), (3) open injury of Achilles tendons, (4) incomplete clinical data or follow-up of less than a year.

Two clinical investigators were responsible for individual data collection. Demographic data (gender and age), injury mechanism, and injury side were obtained from medical records. In addition, surgical details, including surgical time and postoperative length of stays (post-LOS), were also reviewed.

### Operative technique

#### *Minimally invasive repair (lumbar puncture needle and oval forceps)* [16, 18]

**Fig. 1:**
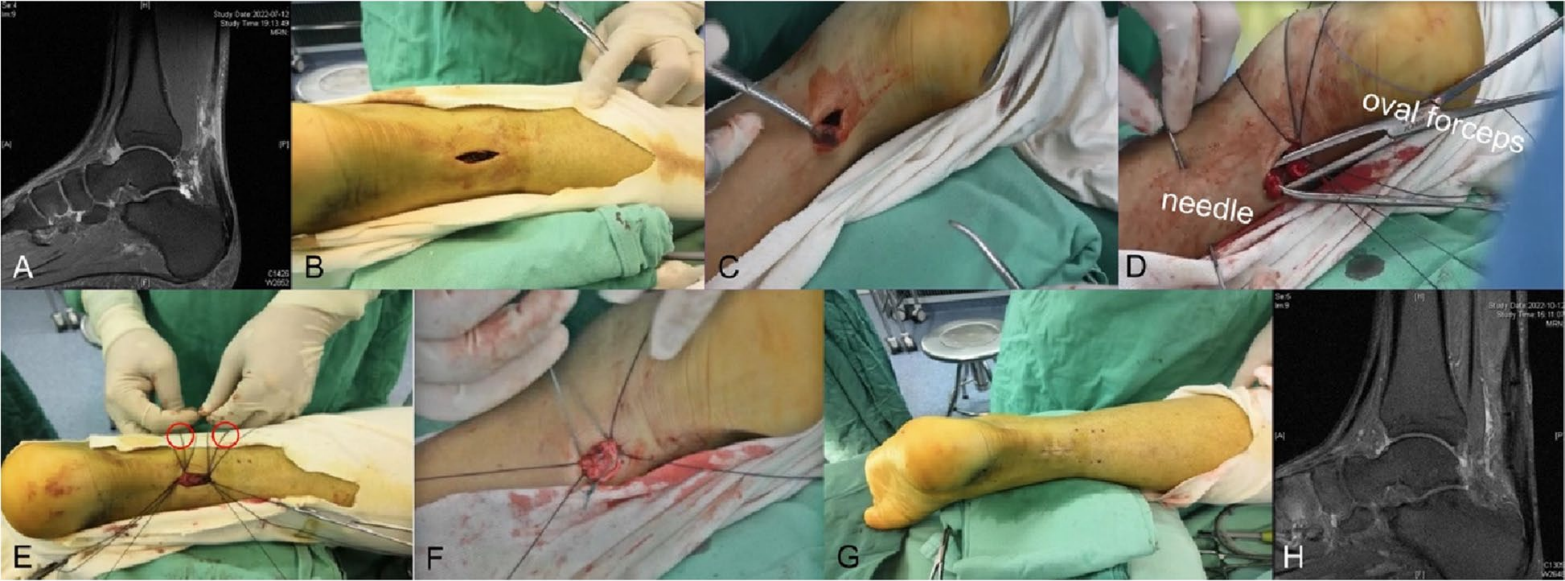
A series of interoperative photos illustrating the minimally invasive Achilles repair. **A** A pre-operative MRI showing a definite rupture of the Achilles tendon. **B** A 2-cm transverse incision was made over the central part of the Achilles tendon defect. **C:** An Allis clamp exposed and retracted the rupture site. **D** After the subcutaneous tunnel was built, the oval forceps was inserted and the Achilles tendon defect was grasped. Then, a lumbar puncture needle was passed through the tendon and the eyelet ring of the oval forceps. **E** Sutures went into the needle. The red circle denoted the nonabsorbable sutures. **F** It should be noted that the stitches were separated to avoid the concentration of suture knots. **G** The wound was sewed up in plantar flexion. **H** Postoperative radiographic data presented that the tendon healed well

The patient was under spinal anesthesia or general anesthesia, in a prone position with a pad under the ankle. (Fig. 1).

(i) The surgeon palpated the tendon’s gap and observed the positive sign (“finger sign”). Then, a 2–3 cm longitudinal incision was immediately made over the ruptured Achilles tendon site.
(ii) An Allis clamp was used to retrieve the proximal/distal broken end. An oval forceps was also inserted to penetrate both sides of the ruptured tendon. A lumber puncture needle (BD Medical System, NJ, USA) created a subcutaneous tunnel through the skin, subcutaneous fat, fascia, paratenon, tendon, and two eyelet rings of the oval forceps. Particularly in confirming whether the needle involved the ruptured tendon, the surgeon should attempt to withdraw the oval forceps.
(iii) Then, a No. 0 polydioxanone (PDS) (Ethicon, Somerville, NJ, USA) was passed through the needle. After the needle and oval forceps were retrieved, the suture tails were concomitantly detached from the incision. Sutures were carefully separated and marked. The above procedure was repeated three times at the proximal/ distal site, and the needle pitch was about 0.8 cm. A No. 2 polyester suture (Ethicon, Somerville, NJ, USA) was used in each middle suture.
(iv) Ruptured tendon strength was tested by gentle retraction of the sutures, which could minimize the risk of entrapping the sural nerve. The operated ankle was located at 30° of plantar flexion during repair. The most proximal suture from the proximal segment was tied to the most proximal from the distal, and so on. The loops were concealed severally beneath the paratenon.
(v) The paratenon/subcutaneous tissue and skin were closed using 2/0 Vicryl (Ethicon, Somerville, NJ, USA) and 3/0 nonabsorbable suture (Ethicon, Somerville, NJ, USA), respectively.A sterile dressing and a plaster splint were applied to the ankle with 30 degrees of plantar flexion.

### Postoperative management

Stage 1: For the first two weeks postoperatively, patients were instructed to apply over-the-knee full casts with 30-degree knee flexion and 30-degree plantar flexion. Additionally, they were allowed to sustain 1/6 to 1/3 of the total weight. Heel cushions were available to adjust the plantar flexion angle and enhance the ankle proprioception.

Stage 2: A below-knee cast and walking brace were used for the next two weeks, and the goal was to gradually reach a full weight bearing. A full range of ankle motion attempts and concentric loading was instituted under the guidance of a physical therapist. Subsequent eccentric loading is gradually carried out according to the patient's recovery.

Stage 3: The walking braces were removed at 5 weeks postoperative, and they could walk with normal shoes. Plyometric exercises were permitted, and patients were encouraged to participate in athletic activities if they felt comfortable during the ninth week.

### Clinical outcomes

To evaluate the operative outcome, we used the Achilles tendon rupture score (ATRS), the American Orthopedic Foot and Ankle Society (AOFAS) ankle/hindfoot score, and the visual analog scale (VAS). The ATRS scoring system is a validated Achilles tendon rupture instrument [23, 24]. The ATRS is a Patient-Reported Outcome Measure (PROM) presenting the difficulty level of various physical activities due to symptoms. The AOFAS scoring systems, including self-assessment and objective scoring, are broadly used to evaluate the ankle joint's clinical function, range of motion, and tolerance [25]. During the follow-up, we also recorded the time to return to work, full weight bearing, and return to previous activities. Furthermore, we collected patient complications during follow-up, including re-rupture, suture reaction, wound dehiscence, and deep infection.

Re-rupture was defined as a definite rupture after surgical repair. Suture reaction and wound dehiscence were counted when the redness/swelling of the incision and superficial wound breakdown, respectively [26]. Deep infection was referred to the previous definition of fracture-related infection, regarding as a postoperative infection presented a year after surgery and met at least one of the pathologic conditions: (1) clinical signs of a fistula, sinus, wound necrosis, or purulent exudation, (2) confirmed microorganisms by laboratory evidence from at least two separate deep tissues or implants during paracentesis or re-operation [27].

### CUSUM analysis

Consecutive cases were enrolled in chronological order. The CUSUM method was applied to analyze the learning curve, focusing on surgical duration [28]. We also used the following formula for the CUSUM calculation: $CUSUM={\sum }_{i=1}^{n}(Xi-U)$*.* 'Xi' was the operation time per case, 'U' was the average operation time across all cases, and 'n' was the consecutive numbering of each case. Python (version 3.9.6) was used for analysis and scatter plot generation was used to obtain the function formula through curve fitting. The efficacy of the curve fitting was assessed using the correlation coefficient R^2^, with values approaching 1 indicating more precise fitting. The first order of the curve was chosen and the curve apex was based on the slope. Notably, this apex contributed to segregating the patients into individual groups of learning and mastery, establishing a cut-off value representing the minimum number of cases in which a surgeon was required to obtain a certain level of proficiency [29].

### Statistical analysis

According to the normal distribution results, continuous variables were analyzed using the independent samples t-test or Mann–Whitney u-test. The enumeration variables were analyzed using the chi-square or Fisher exact tests where appropriate. Descriptive data were presented as mean ± standard deviation (SD), median (interquartile range, IQR), and frequency (percentage) for normally and non-normally distributed continuous data and enumeration data. All statistical analyses were performed using SPSS software (IBM SPSS Statistics, Version 26.0). A *p*-value of < 0.05 was considered statistically significant in this study.

## Results

### Baseline data and learning curve quantification

**Fig. 2:**
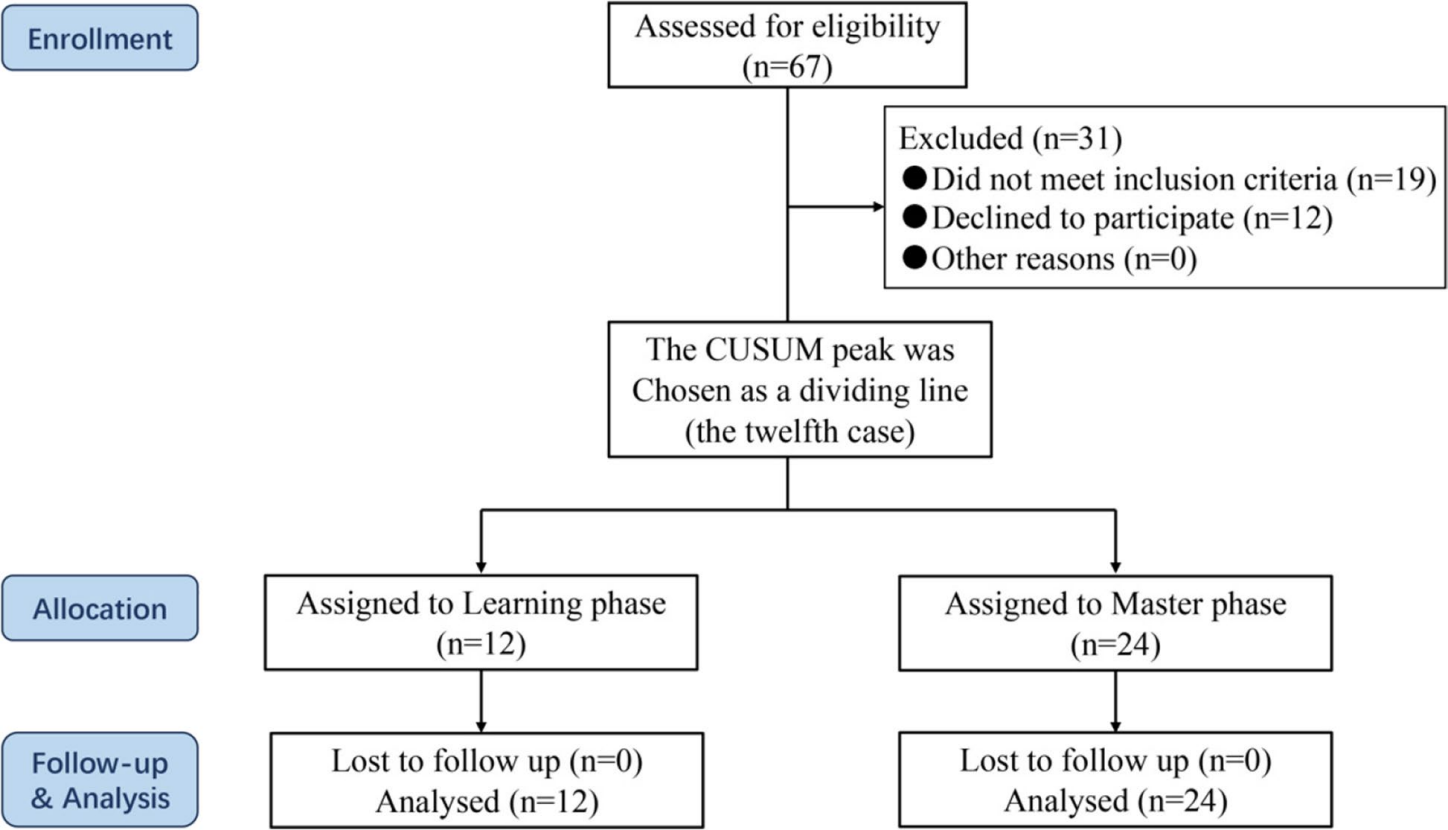
Flowchart of the study design

In general, a consecutive cohort of sixty-seven cases who received a “lumbar puncture needle and oval forceps” in treating acute Achilles rupture in our institution were reviewed. Finally, thirty-six patients were enrolled in this study (Fig. 2).

**Fig. 3:**
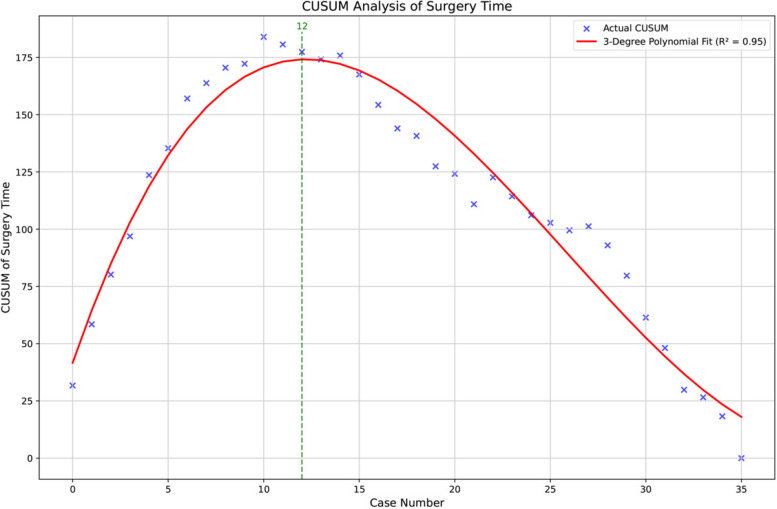
The cumulative sum curve for surgical time: peak at the twelfth case

Since these results of surgical performance demonstrated that the learning curve peaked at the 12th case, we divided the cases into 2 groups: the learning phase (*n *= 12, case 1–12) and master phase (*n* = 24, case 13–36) (Fig. 3). The best-fit linear equation was: CUSUM = 1.68 × 10^−2^n^3^—1.30 × 10^0^n^2^ + 2.42 × 10^1^n + 4.14 × 10^1^ (R^2^ = 0.95), where n represented the individual case.

**Fig. 4:**
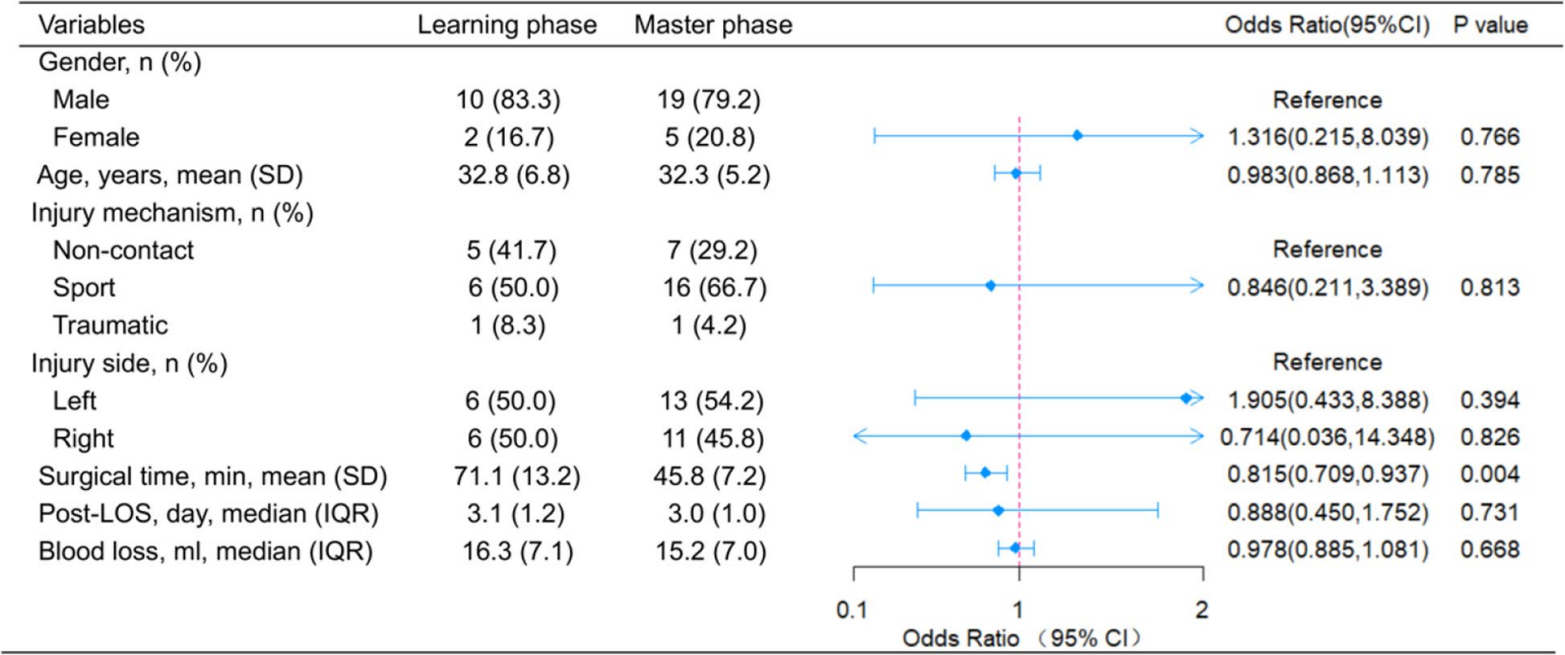
The forest graph illustrating the comparisons of baseline data and surgical details between the learning and master phases

The learning phase and master phase were statistically similar in gender ratio (the learning phase 83.3%, the master phase 79.2%, *p* = 0.766) and age (the learning phase 32.8 ± 6.8 years, the master phase 32.3 ± 5.2 years, *p* = 0.785) (Fig. 4). Other clinical data also demonstrated no statistical difference between these groups in injury mechanism, injury side, post-LOS, and blood loss (*p* > 0.05). However, the learning phase was significantly longer than the master phase for surgical time (71.1 ± 13.2 vs. 45.8 ± 7.2 min, *p* = 0.004).

### Postoperative assessments

**Fig. 5:**
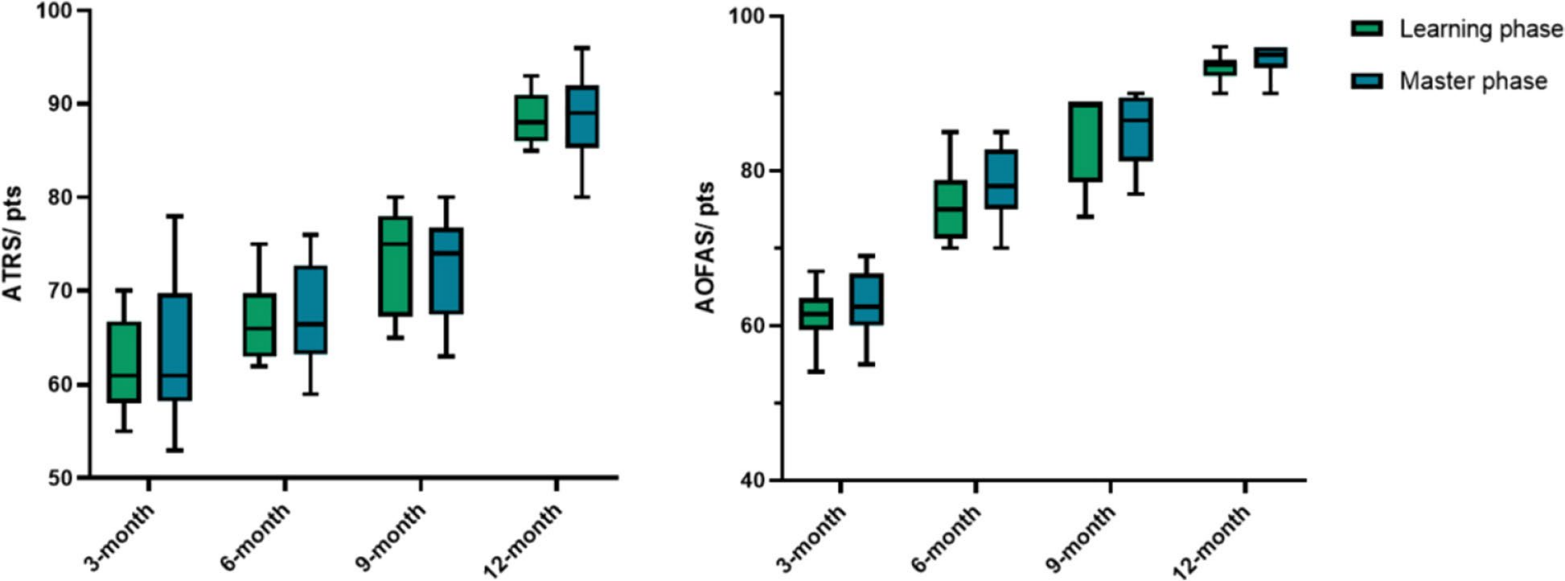
Boxplots of the clinical scoring systems between the two groups. The horizontal lines, the boxes and the whiskers represent the median scores, the interquartile range and the minimum/ maximum, respectively

**Fig. 6:**
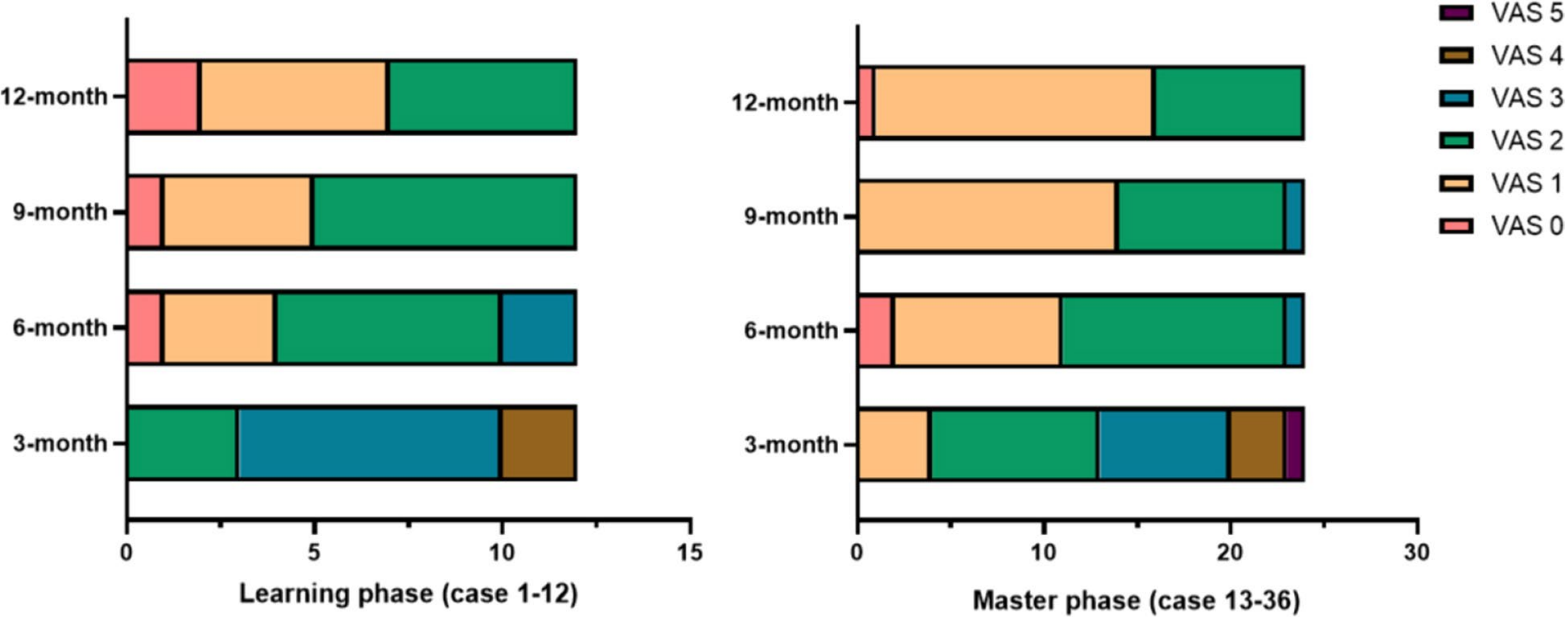
Proportion bar charts of VAS in these groups at 3/6/9/12 months

The clinical outcome evaluation was assessed using the ATRS and AOFAS. The VAS scoring system determined the pain. We found no statistical correlation between these ATRS, AOFAS, and VAS groups in 3/6/9/12 months (Table 1, Figs. 5 and 6). Similarly, neither time to return to work/ full-weight bearing/ previous activities showed a significant difference (*p* > 0.05).

**Table 1:** Follow-up data comparison between the learning phase and master phase

Variable	Learning phase	Master phase	*p* value
ARTS, pts, mean ± SD			
3-month	62.3 ± 5.1	63.3 ± 7.0	0.68
6-month	66.8 ± 4.4	67.6 ± 5.1	0.61
9-month	73.2 ± 5.7	72.3 ± 5.7	0.65
12-month	88.3 ± 2.9	88.7 ± 4.6	0.80
AOFAS, pts, mean ± SD			
3-month	61.2 ± 3.3	62.7 ± 4.1	0.32
6-month	75.8 ± 5.1	78.7 ± 4.6	0.09
9-month	84.3 ± 6.0	85.2 ± 4.3	0.58
12-month	93.5 ± 1.7	94.5 ± 1.8	0.12
VAS, pts, mean ± SD			
3-month	2.9 ± 0.7	2.5 ± 1.0	0.22
6-month	1.8 ± 0.9	1.5 ± 0.7	0.37
9-month	1.5 ± 0.7	1.4 ± 0.6	0.85
12-month	1.3 ± 0.8	1.3 ± 0.6	0.85
Time to return to work, weeks, mean ± SD	8.1 ± 1.2	7.9 ± 1.1	0.67
Time to full weight bearing, weeks, mean ± SD	8.8 ± 1.2	8.4 ± 1.2	0.28
Time to return to previous activities, months, mean ± SD	4.9 ± 0.8	5.0 ± 0.8	0.66

*ATRS* Achilles tendon rupture score; *AOFAS* American Orthopedic Foot and Ankle Society; *VAS* visual analogue scale; *SD* standard deviation

Regarding the complication within a year, one patient (case 4) was detected with a suture reaction, which presented in the fourth month postoperatively. After removing the nonabsorbable sutures, the redness and swelling of his injured Achilles tendon gradually improved in the following seven days. Major complications, including re-rupture, wound dehiscence, and deep infection, were not found in these thirty-six patients.

## Discussion

Achilles tendon rupture is a common injury in daily life, accounting for 35% of the rate of human tendon damage. This injury always occurs during motor activity, of which men aged 30 – 50 are the most susceptible [30, 31]. A clinical study found that the male proportion and the mean age of the entire study were 88.9% and 36.9 years, consistent with our research [32]. We also detected that patients were keen on exercise and usually in good physical condition before the tendon got hurt. For such patients, they were enthusiastic about rehabilitation.

Despite it usually suddenly occurring, however, there is still a fair amount of chronic Achilles tendon rupture that is ascribed to misdiagnosis or disregarding this injury. Chronic Achilles tendon rupture mainly presents with cicatricial tissues irregularly buttressing the rupture gap and causing broken ends retraction, gastrocnemius atrophy, and gait incoordination [33, 34]. Consequently, these uncertainties and diversity produce different therapeutic regimens compared to acute injuries. Therefore, we excluded patients with chronic Achilles tendon rupture from this study.

Many different techniques have been introduced for operative interventions. Previous research pointed out that open surgery had a deficiency in wound healing and deep infection [35–37]. Although the percutaneous method minimized the wound complications, it increased the risk of the sural nerve injury and re-rupture [38]. A cadaveric study also observed that a minimally invasive method could be safe for sural nerves if the technique were used correctly [11, 39]. Campillo-Recio et al. disagreed that the percutaneous method was superior to the conservative treatment because the latter allowed the earlier weight-bearing and controlled rehabilitation protocol at a young age [40]. Furthermore, Attia et al. reported that the incidence of deep infection for open surgery was over 20% [41]. However, our results did not detect deep infection, possibly due to the small number of patients we included and the short follow-up period.

In 2014, a technique was described that included a mini-open surgery combined with a knotless percutaneous instrument (the Achilles Midsubstance SpeedBridge) to repair the injured site [42]. Hoskins et al. also reported that patients treated by an Arthrex PARS Achilles Jig System and their postoperative AOFAS and ATRS reached 90.3 and 88.0 points [9]. Concerning complications, they observed cases of re-rupture (1.2%) and suture irritation (1.2%). Notably, these follow-up data were similar to ours. Thus, patients could obtain a full weight bearing in the ninth week after surgery, conforming to the principle of rehabilitation.

For economic reasons, our institution did not import these instruments. Therefore, we referred to previous studies and used the simplified instrument, “the oral forceps and lumbar puncture needles.” This method was first reported by Ngai et al. and significantly reduced the surgery cost, which was widely used worldwide [18]. Liu et al. inserted an additional two anchors into the calcaneus from this previous study. However, we thought this also caused unnecessary waste [16]. Thus, our study used only three sutures and ensured these knots were staggered to avoid tissue cutting and centralization. Follow-up results also showed a relatively clinical outcome. Biz et al. used Tenolig technology to repair the Achilles tendon rupture, which cost nearly 1,000 euros [43].

In this study, the average operative time was 45.8 min after reaching mastery. Attia et al. included ten randomized controlled studies on open versus minimal invasive acute Achilles tendon rupture repair. The results showed that the average time was 51.0 and 29.7 min [41]. We took a longer surgical time for each procedure, which might be attributed to different calculations, defined as the time interval from surgical area disinfection to wound closing in our institution.

We recognize that there are several limitations to this study. Firstly, the relatively small series size, including its retrospective design, limits the reliability of our research. A power analysis was not conducted, and our results must be interpreted cautiously. Secondly, this minimally invasion surgery was performed by an individual surgeon. Discrepancies in the learning curves exist among surgeons, so its generalization remains unknown. For all this, our study has proved surgeons could be proficient with this technique over time. However, additional studies are needed to support these findings.

## Conclusions

Our learning curve analysis demonstrated that the surgical competence in treating an acute Achilles tendon rupture with the “lumbar puncture needle and oval forceps” was obtained after the primary learning period of 12 cases. Furthermore, this technique benefited early rehabilitation and rapid return to normal life.

## Data Availability

The datasets used and/or analyzed during the current study are available from the corresponding author on reasonable request.
